# Blood disorders typically associated with renal transplantation

**DOI:** 10.3389/fcell.2015.00018

**Published:** 2015-03-19

**Authors:** Yu Yang, Bo Yu, Yun Chen

**Affiliations:** ^1^Department of Urology, First Affiliated Hospital of PLA General HospitalBeijing, China; ^2^BrightstarTech, Inc.Clarksburg, MD, USA

**Keywords:** renal transplantation, post-transplant blood disorders, immunosuppressive agents, viral infections, bone marrow suppression, impaired hematopoiesis, clinical features, therapeutic strategy

## Abstract

Renal transplantation has become one of the most common surgical procedures performed to replace a diseased kidney with a healthy kidney from a donor. It can help patients with kidney failure live decades longer. However, renal transplantation also faces a risk of developing various blood disorders. The blood disorders typically associated with renal transplantation can be divided into two main categories: (1) Common disorders including post-transplant anemia (PTA), post-transplant lymphoproliferative disorder (PTLD), post-transplant erythrocytosis (PTE), and post-transplant cytopenias (PTC, leukopenia/neutropenia, thrombocytopenia, and pancytopenia); and (2) Uncommon but serious disorders including hemophagocytic syndrome (HPS), thrombotic microangiopathy (TMA), therapy-related myelodysplasia (t-MDS), and therapy-related acute myeloid leukemia (t-AML). Although many etiological factors involve the development of post-transplant blood disorders, immunosuppressive agents, and viral infections could be the two major contributors to most blood disorders and cause hematological abnormalities and immunodeficiency by suppressing hematopoietic function of bone marrow. Hematological abnormalities and immunodeficiency will result in severe clinical outcomes in renal transplant recipients. Understanding how blood disorders develop will help cure these life-threatening complications. A potential therapeutic strategy against post-transplant blood disorders should focus on tapering immunosuppression or replacing myelotoxic immunosuppressive drugs with lower toxic alternatives, recognizing and treating promptly the etiological virus, bacteria, or protozoan, restoring both hematopoietic function of bone marrow and normal blood counts, and improving kidney graft survival.

Renal transplant or kidney transplant is surgical procedure to place a functioning kidney from a donor into a patient with kidney failure. A renal transplant is often the best treatment option for patients with end stage kidney failure. The successful renal transplant could offer enhanced quality and duration of life, which make the recipients return to a more normal lifestyle and have more control over their daily living. With the advent of new immunosuppressive drugs and improved donor-recipient matching procedures, rates of serious complications following renal transplantation have fallen sharply in recent years. However, like any other type of surgery, kidney transplant surgery carries a risk of significant complications. Some blood disorders may occur and develop after renal transplantation. Common post-transplant blood disorders include post-transplant anemia (PTA), post-transplant lymphoproliferative disorder (PTLD), post-transplant erythrocytosis (PTE), and post-transplant cytopenias (PTC, including leucopenia, thrombocytopenia, and pancytopenia). Uncommon but serious blood disorders are hemophagocytic syndrome (HPS), thrombotic microangiopathy (TMA), therapy-related myelodysplastic syndromes (t-MDS), and therapy-related acute myeloid leukemia (t-AML) (Ardalan et al., 2008; Reindl-Schwaighofer and Oberbauer, 2014). Understanding how these post-transplant blood disorders occur and develop in renal transplant recipients is critical for developing further therapeutic strategies for these life-threatening hematological complications. In this review, we discuss the etiology, pathogenetic mechanisms, clinical features and current management of each blood disorder mentioned above.

## Post-transplant anemia (PTA)

PTA, as a common complication after renal transplantation, is attracting more and more attention of urological community (Augustine and Hricik, 2006). It is usually classified as acute (within 6 months post-renal transplant) and chronic (more than 6 months post-renal transplant). PTA may persist or reoccur after renal transplantation. It occurred at least once in 38.3%, and reoccurred in 42% of renal transplant recipients within 5 years (Yu et al., 2010). PTA can be caused by several conditions in renal transplant recipients, including renal allograft dysfunction, medications [immunosuppressive agents, angiotensin-converting enzyme (ACE) inhibitors, angiotensin II receptor antagonists, and antiviral and antimicrobial medications], viral infections, acute rejection, nutritional deficiency, and blood group ABO incompatibility (Reindl-Schwaighofer and Oberbauer, 2014).

Post-transplant renal allograft dysfunction has been recognized as a major cause of PTA. The incidence of PTA, which increases over time after the transplants, is positively related to the level of creatinine in the blood. When the serum creatinine level rises above 2 mg/dL, a poorly functioning kidney allograft will result in reduced erythropoietin (EPO) production. The higher the serum creatinine level, the higher the incidence of PTA (Imoagene-Oyedeji et al., 2006). The EPO level is usually consistent with kidney functions after the transplants. As renal function deteriorates, renal EPO secretion will decrease, causing PTA in renal transplant recipients. Anemia related to chronic kidney disease (CKD) is characterized by loss of endogenous EPO production and/or EPO hyporesponsiveness/resistance in the patients (Winkelmayer and Chandraker, 2008). In some patients with delayed graft function (DGF) after the transplants, an increase in EPO level can be observed. Such an increase is generally not accompanied by an increased in hemoglobin level, and may return to its original level in a short time. The EPO level is negatively correlated with the hemoglobin levels, suggesting EPO hyporesponsiveness/resistance in patients with PTA (Rostami et al., 2012).

Chronical exposure to immunosuppressive agents has been known to be an important factor in the development of anemia after transplantation, which operates mainly through direct suppression of erythropoiesis in bone marrow (Winkelmayer and Chandraker, 2008). Immunosuppressant agents include antiproliferative agents [such as azathioprine (AZA), mycophenolate mofetil (MMF), thiopurine methyltransferase (TPMT), and sirolimus], calcineurin inhibitors [such as cyclosporine (CsA) and tacrolimus (FK-506)], induction immunosuppressive agents [such as muromonab-CD3 (OKT3)], and anti-thymocyte globulin (ATG) (Nguyen and Shapiro, 2014). A standard immunosuppressive regimen in renal transplantation consists of combination therapy with an antiproliferative agent, an induction immunosuppressive agent, a calcineurin inhibitor, and a corticosteroid. These drugs may result in an increased incidence of PTA when they are used after the transplants (Al-Khoury et al., 2006).

Among the immunosuppressive agents, the antiproliferative agents (AZA and MMF) and the calcineurin inhibitor (FK-506) are myelosuppressive drugs that cause potentially the suppression of bone marrow. PTA caused by these myelotoxic drugs is generally associated with leukopenia and/or thrombocytopenia (Lee et al., 2009). Calcineurin inhibitors may indirectly cause PTA via nephrotoxicity, microangiopathic hemolytic anemia (MAHA) and/or hemolytic-uremic syndrome (HUS) (Vergoulas, 2006). Nephrotoxicity may occur after the application of a calcineurin inhibitor that causes damage to the kidneys. This condition will result in elevated blood levels of urea nitrogen (BUN), creatinine, and electrolytes (such as potassium and magnesium) (Paige and Nagami, 2009). MAHA results from platelet aggregation and fibrin deposition in the damaged endothelium of small vessels to form a fibrin mesh. The fibrin mesh causes narrowing or obstruction of small blood vessels and increases shear forces within these small vessels, thus leading to mechanical destruction (intravascular fragmentation hemolysis) of the red blood cells and schistocyte formation (Korin et al., 2012). The immunosuppressant OKT3 has been associated with MAHA (Kim et al., 2011). HUS is a condition resulting from destruction of red blood cells that clogs the filtering system in the kidneys. *De novo* HUS occurs often associated with the use of CsA or OKT3 (Vergoulas, 2006), and may subsequently induce hypertension, MAHA, TMA, and end-stage renal failure (Ardalan, 2006). Sirolimus is a non-nephrotoxic macrolide antibiotic that reduces cytokine-dependent cellular proliferation at the G1 to S phase of the cell-division cycle. It may inhibit erythropoiesis by interfering with intracellular signaling pathways normally activated after the binding of erythropoietin to its receptor, resulting in anemia, thrombocytopenia, and leucopenia (Fang et al., 2007).

In addition, immunosuppressant drugs induce increased susceptibility to virus such as parvovirus B19 (PVB19), cytomegalovirus (CMV), and Epstein-Barr virus (EBV) in renal transplant recipients, which cause aplastic anemia (Egbuna et al., 2006). CMV and PVB19 infections have been reported in transplant recipients with PTA and may cause pure red cell aplasia (PRCA) (Pinto et al., 2008). Viral infections (CMV, EBV, influenza A, and human heprus virus 6 and 8) can also cause HPS (a rare cause of PTA) and HUS in transplant recipients (Asaka et al., 2000; Maakaroun et al., 2010). Some immunosuppressive agents (such as AZA, MMF, and FK-506) cause not only the suppression of bone marrow, but also erythroblastopenia (PRCA) (Geetha et al., 2000). PRCA is an uncommon blood disorder in which RBC maturation arrest occurs in the bone marrow. Erythroblasts are virtually absent, however, WBC and platelet production is normal in bone marrow. The anemia due to PRCA is a severe anemia, in which a reticulocyte count is generally less than 1%, and mature erythroblasts present less than 0.5% in the bone marrow (Fallahi et al., 2014). PRCA can develop in patients with PVB19 infection (Kurukulasuriya et al., 2011; Baral et al., 2012), erythropoietin therapy (Casadevall et al., 2002; Macdougall et al., 2012), ABO-incompatible transfusion (Rowley et al., 2011), and stem cell transplantation (Stussi et al., 2009). PVB19 may cause PRCA due to its viral tropism and direct cytotoxicity to erythroid progenitor cells (Heegaard and Brown, 2002). PRCA can also be a result of therapy with certain recombinant forms of EPO, probably because of production of neutralizing antibodies that inhibit the erythropoietic activity of endogenous EPO and recombinant erythropoiesis-stimulating agents (ESAs) (Casadevall, 2005). PRCA can further develop into aplastic anemia and acute myelogenous leukemia, which are life threatening illnesses for patients (Clark et al., 1984). High-dose intravenous immunoglobin therapy should be considered for PRCA caused by PVB19 infections and erythropoietin therapy. PRCA may be reversible by immunoglobulin within a few months (Kurukulasuriya et al., 2011; Baral et al., 2012).

Both ACE inhibitors and angiotensin II (ATII) receptor antagonists have an inhibitory action on the generation of red blood cells in renal transplant patients. The use of these drugs in renal transplant recipients can cause PTA, especially in the patients with post-transplant renal failure (Chhabra et al., 2008). The mechanisms by which ACE inhibitors and ATII receptor antagonists cause PTA, include inhibition of endogenous erythropoietin production, inhibition of ATII-mediated stimulation of red blood cell precursors (Macdougall, 1999), and the generation of an erythropoiesis-inhibiting protein by ACE inhibitors (Henriksen and Jacob, 2003). In addition, the application of antiviral (Chhabra et al., 2008), antimicrobial (Reindl-Schwaighofer and Oberbauer, 2014), and antilymphocyte (Jordan et al., 1999) drugs [such as ganciclovir, trimethoprim-sulfamethoxazole, antilymphocyte globulin (ALG)] have been reported to cause PTA in renal transplant recipients.

PTA can also be caused by transplant rejection and nutritional deficiencies. Acute rejection results in a sharp decrease in erythropoietin production and anemia at the early stage of renal transplantation (Galutira and Del Rio, 2012). Transplant rejection also causes systemic inflammatory response syndrome in renal transplant recipients. It disturbs the binding of iron to folic acid and blocks their transport, thus leading to PTA. In pediatric renal allograft recipients with acute rejection, an “erythropoiesis cluster” of 11 genes involved in hemoglobin (Hb) transcription and synthesis, iron and folate binding, and transport were found to be downregulated (Chua et al., 2003). TMA that may develop during episodes of severe vascular rejection, is another mechanism for the development of PTA during rejection (Barbour et al., 2012). Once acute rejection is controlled, the EPO secretion can be restored to normal level. Post-transplant nutritional deficiencies such as iron deficiency, folic acid deficiency, and vitamin B6/B12 deficiency result in PTA due to a lack of nutrients needed for blood cell production (Chadban et al., 2010). PTA caused by iron deficiency is generally associated with blood loss during dialysis or the operative procedure, excessive collection of blood samples prior to transplantation, abnormal coagulation related to renal failure, and post-operative gastrointestinal bleeding (Galutira and Del Rio, 2012).

Blood group ABO-incompatible transplantation is a medical technique of allocating human organs for transplantation. This innovative desensitization technique can eliminate a recipient's reaction to an incompatible blood type, and permits more efficient use of available organs regardless of ABO blood type that will otherwise be unavailable due to hyperacute rejection (West et al., 2006). However, ABO-incompatible transplantation can cause significant hemolysis or hemolytic anemia in transplant recipients. Hemolysis following ABO-incompatible transplantation is caused by a type of graft-vs.-host reaction in which the B-lymphocytes in the donor organ produce ABO antibodies (typically immunoglobulin G) to the ABO antigens of the recipient. Hemolysis occurs more often in the blood group type A recipients (Maheshwari et al., 2004). ABO-incompatible kidney transplantation is also associated with PTLD and renal failure (Nose et al., 2012).

Recent studies have shown that PTA is commonly associated with post-transplant CKD or called chronic allograft nephropathy (CAN) in renal transplant recipients. It might begin to develop in the early stages of CKD, and might tend to worsen as CKD progresses (Brugnara and Eckardt, 2011; Choukroun et al., 2012). In addition, PTA is also probably the most common risk factor that causes cardiovascular diseases (such as *de novo* congestive heart failure and left ventricular mass) and the death of renal transplant recipients (Winkelmayer and Chandraker, 2008). Therefore, appropriate treatment for PTA is important for slowing post-transplant CKD progression and preventing cardiovascular complications in renal transplant recipients.

The goals of therapy for PTA should be to restore EPO production, to maintain hemoglobin at an adequate level, to enhance kidney graft survival, and to treat underlying cardiovascular disorders (Malyszko et al., 2012). ESAs are recommended to treat anemia resulting from chronic kidney failure, chemotherapy, certain treatments for Human Immunodeficiency Virus (HIV), and also to reduce the number of blood transfusions during and after certain major surgeries (Hung et al., 2014). ESAs include EPO, epoetin alfa (Procrit/Epogen), epoetin beta (NeoRecormon), darbepoetin alfa (Aranesp), and methoxy polyethylene glycol-epoetin beta (Mircera). These agents work by stimulating the bone marrow to produce red blood cells and maintaining hemoglobin at the lowest level. ESAs have been proven effective at correcting PTA after renal transplantation (Locatelli and Del Vecchio, 2011). Correction of PTA with ESAs (such as EPO and epoetin beta) has a beneficial effect on slowing post-transplant CKD progression and improving quality of life in renal transplant recipients. The application of high doses of ESAs in renal transplant recipients can obviously improve physiological function and glomerular filtration rate (GFR) of the grafted kidneys, thus reducing the incidence of endstage renal disease and enhancing long-term kidney graft survival (Choukroun et al., 2012).

## Post-transplant lymphoproliferative disorder (PTLD)

PTLD is a well-recognized complication of both solid organ transplantation (SOT) and allogeneic hematopoietic stem cell transplantation (HSCT). In renal transplant recipients, PTLD is usually caused by EBV infection due to therapeutic immunosuppression after renal transplantation (Newstead, 2000). EBV is a herpes virus that latently infects B-cell lymphocytes and chronically replicates in the cells of the oropharynx (Comoli et al., 2005). In normal condition, EBV infection activates innate immunity (both humoral and cellular immunity) to control EBV replication and proliferation of EBV-infected B-cells. However, when immunosuppressants such as FK-506 and CsA are used to prevent graft rejection after renal transplantation, these drugs will impair CD4 and CD8 T-cell immunity by inhibiting T cell function, thus leading to uncontrolled proliferation of EBV-infected B-cells to form lymphoid hyperplasia or B cell lymphoma (Kamaleshwaran et al., 2014). Depletion of T cells by use of anti-T cell antibodies (ATG, ALG, and OKT3) to prevent or treat transplant rejection may also increases the risk of developing PTLD.

The molecular pathogenesis of PTLD caused by EBV infection may involve genetic or epigenetic alterations of several cellular genes, such as translocations (involving C-MYC, IGH, BCL-2, BCL-6), various copy number variations, DNA mutations (PIM1, PAX5, p53, C-MYC, RhoH/TTF), DNA hypermethylation, and polymorphisms in both the host cell's genome (IFN-gamma, IL-10, TGF-beta, HLA) and the EBV genome (Capello and Gaidano, 2009). As a consequence of defects in the DNA mismatch repair mechanism, microsatellite instability in the tumor microenvironment seems to play an important role in the development of PTLD, which allows antitumor immune responses even in an immunocompromised host (Capello et al., 2005; Morscio et al., 2013).

Most cases of PTLD usually occur with 1–2 months of organ transplant. The incidence rate of PTLD varies between 1 and 5% in renal transplant recipients (Preiksaitis, 2004). There is an increased risk of PTLD in the patients with a negative EBV-specific test result before kidney transplantation, as compared to those with a positive EBV-specific test result. In addition, if renal transplantation is performed in children less than 5 years of age, a marked increase in the incidence of PTLD can be observed (Allen et al., 2001). PTLD is often aggressive, rapidly progressive, and potentially life threatening. Its related mortality ranges from 25 to 26.6% (Wasson et al., 2006). The patients with PTLD may develop infectious mononucleosis-like lesions or polyclonal polymorphic B-cell hyperplasia (Zimmermann and Trappe, 2011). PTLD often spread to organs or tissues (the lung, gastrointestinal tract, and central nervous system) outside of the lymph nodes. Clinical presentation of PTLD is variable and includes: an acute infectious mononucleosis-like disease with marked constitutional upset and rapid enlargement of tonsils and cervical nodes, a fulminant presentation that presents with widespread infiltrative disease with multi-organ involvement, isolated or multiple tumors involving the allograft, gastrointestinal symptoms, and pulmonary nodules (Loren et al., 2003).

The typical histopathological changes of PTLD could be a large increase in the number of B cell lymphocytes in lymphoid tissues, accompanied by multiple focal areas of necrosis (Elmore, 2006). The lesion may include plasmacytic hyperplasia, B-cell hyperplasia, B-cell lymphoma, or immunoblastic lymphoma. Histopathological evidence for the presence of EBV DNA, RNA, or protein is very important to make an accurate diagnosis of PTLD, and can be obtained by using immunohistologic staining of paraffin-embedded tissue, *In situ* hybridization with the EBV-encoded RNA probe, and qualitative and quantitative EBV polymerase chain reaction (PCR) (Gulley, 2001). Qualitative and quantitative EBV PCR is the most commonly used laboratory test for the monitoring of the EBV viral load in the peripheral blood of the recipients and for the diagnosis of PTLD after renal transplantation. EBV viral load measurement in peripheral blood mononuclear cells (PBMCs) and serum samples using quantitative and quantitative PCR techniques has been proven to be a powerful diagnostic tool to monitor transplanted patients at risk to develop PTLD (Merlino et al., 2003). Because healthy transplant recipients usually have low EBV viral loads in the peripheral blood, high EBV loads may be strongly associated with current or impending PTLD (Gulley and Tang, 2010). A trend of EBV PCR values changed over time is more valuable in making a reliable diagnosis of PTLD. Declines in EBV loads may be associated with response to therapeutic interventions (such as immunosuppression tapering, rituximab therapy, and chemotherapy) (Tsai et al., 2002).

PTLD may spontaneously regress with tapering immunosuppressive therapy (reduction or cessation of immunosuppressive treatment). Immunosuppression tapering can lead to hematological remission and attenuation of symptoms associated with PTLD (Karras et al., 2004). However, immunosuppression tapering may not always have benefits to the renal transplant recipients because reduction of immunosuppression increases the risk of inducing allograft dysfunction or loss. Other potential treatments include (1) immunomodulator agent (rituximab), (2) passive immunization with anti-EBV monoclonal antibodies (anti-B-cell monoclonal antibody, anti-CD21 antibody, or anti-CD24 antibody), (3) antiviral therapy against CMV (acyclovir, valacyclovir, ganciclovir, or foscarnet), (4) interferon alfa-2b (Intron A) therapy, (5) T-cell–based therapy (EBV-specific cytotoxic T lymphocytes), (6) intravenous gamma globulin (IVIG) therapy (gamimune, gammagard S/D, sandoglobulin), (7) antineoplastic agents (prednisone, cyclophosphamide, doxorubicin, vincristine,etc.), (8) combination chemotherapy (rituximab followed by cyclophosphamide, adriamycin, oncovin, and prednisone), (9) surgery excision, and (10) localized radiation therapy (Weikert and Blumberg, 2008).

Rituximab, an IgG1 kappa immunoglobulin, is an engineered chimeric murine/human monoclonal antibody that contains murine heavy and light chain variable regions directed against both the CD20 antigen found on pre-B lymphocytes and mature B lymphocytes and the human IgG1 constant region. It can block proliferating, apoptosis and lysis of B lymphocytes through complement-dependent cytotoxicity, antibody-dependent cell cytotoxicity, and activation of tyrosine kinases as a direct effect of the antibody binding to its CD20 ligand (Jain et al., 2005). Rituximab has been successfully used in the renal transplant setting, to facilitate desensitization in both highly sensitized transplant recipients and ABO-incompatible transplant recipients, to treat CD20-expressing PTLD following renal transplantation, to treat rejection associated with B cells and antibodies, and to treat recurrent glomerular diseases in the renal allograft (Barnett et al., 2013). Recent evidence indicates that rituximab might be a safe and effective agent to treat PTLD, with generally an overall response rate of 50–69% (Becker et al., 2006; Salama and Pusey, 2006; Kahwaji et al., 2009). Interferons can modulate the response of the immune system to EBV, control B cell proliferation, and promote TH1 response to virus. Anti-EBV monoclonal antibodies may slow down the proliferation of B cell lymphocytes. Combination chemotherapy with rituximab followed by CHOP (cyclophosphamide, adriamycin, oncovin, and prednisone) is efficacious in the management of PTLD and may be superior to rituximab monotherapy followed by chemotherapy at the time of PTLD progression or relapse. The therapies mentioned above may be effective in preventing clinical deterioration in the condition of patients with PTLD (Martorelli et al., 2012). Recently, a phase 2 study of adoptive transfer of EBV-specific T cells has demonstrated high efficacy with minimal toxicity (Louis et al., 2010).

## Post-transplant erythrocytosis (PTE)

PTE is another common complication after renal transplantation. It is defined as persistently elevated hemoglobin (Hb) (>17 g/dL) and hematocrit (>51%) levels and persists for more than 6 months (Einollahi et al., 2005; Kiberd, 2009). A significant increase in hemoglobin and hematocrit levels will leads to increased viscosity of the blood, causing thrombosis, hypertension and other complications (e.g., cerebral vascular accident) in renal transplant recipients. PTE occurs in 10–20% of renal transplant recipients and usually develops 8 to 24 months after the transplants. Like other forms of erythrocytosis, the patients with PTE often experience malaise, headache, plethora, lethargy, dizziness, and thromboembolism. Approximately 1–2% of patients eventually die from complications related to PTE (Vlahakos et al., 2003).

Multiple factors may be involved in the development of PTE. Considerable evidence indicates that at least three hormonal systems [EPO, the renin-angiotensin system (RAS), and endogenous androgens] participate in the pathogenesis of PTE (Vlahakos et al., 2003). EPO is an essential glycoprotein hormone that controls erythropoiesis (red blood cell production). It is mainly produced by interstitial fibroblasts in the kidney in close association with peritubular capillary and tubular epithelial tubule. In many cases of PTE, over-production of EPO is provoked by the transplanted kidney. Endogenous EPO-release from the kidneys stimulates erythropoiesis (Jelkmann, 2011). Some factors have been suggested to be involved in over-production of EPO after the transplants, which include chronic rejection, renal artery stenosis, cyclosporin, and hydronephrosis of the transplanted kidney. These factors have been shown to cause PTE by inducing renal ischemia and over-production of EPO (Abdelrahman et al., 2004).

The RAS could be involved in the pathogenesis of PTE in renal transplant recipients. RAS is a hormone system that regulates blood pressure and erythropoiesis. Angiotensin II might be responsible for inappropriately sustaining secretion of erythropoietin through directly stimulating the erythroid progenitors within the bone marrow or augmenting the production of other erythropoietic factors, thus increasing erythropoiesis. Enhanced erythropoiesis can also be mediated by activation of the RAS via angiotensin II AT1 receptor of kidney cells (Kato et al., 2005; Park and Zambidis, 2009). Male gender is one important factor predisposing to PTE. Androgens enhance sensitivity of primitive erythrocytes to EPO, activating increased erythropoiesis in male patients. This is the reason why PTE develops more frequently in male patients than females (Yildiz et al., 2003).

Some non-EPO erythropoiesis-stimulating factors may either enhance the sensitivity to EPO or directly promote erythropoiesis. An anomaly in insulin-like growth factor-1 (IGF-1) and its binding proteins-1 and -3 have been accused to induce PTE (Malyszko et al., 2012). The oligopeptide N-acetyl-seryl-aspartyl-lysyl-proline (Ac-SDKP) is a natural inhibitor of hematopoietic stem cell proliferation which is degraded mainly by ACE. Once ACE is inhibited, Ac-SDKP will accumulate in plasma and cause a decrease in erythropoiesis (Chen et al., 1992). After the transplants, the kidney will start removing toxins from the body, which progressively reduce bone marrow suppression caused by the toxins. This may enhance hematopoietic function of bone marrow and cause an increase in red blood cell production (Gaciong et al., 1996). Other causes for an increased erythropoiesis, may include smoking, overnutrition, hypertension, and diabetes (Ahmed et al., 2012).

The treatment goal for PTE is to lower Hb level below 17.5 g/dl in normotensive patients. PTE can be effectively treated with the RAS inhibitors including ACE inhibitors (such as captopril, enalapril, lisinopril, fosinopril, peridropril, and ramipril), and angiotensin II receptor antagonists (losartan, candesartan, telmisartan, and valsartan) (Cruzado et al., 2008). Several other drugs also showed efficacy in treatment of PTE in renal transplant recipients: theophylline, a nonselective adenosine receptor antagonist, can be used as an alternative mode of therapy of PTE; ketanserin, an antagonist of peripheral 5-HT2 receptors, can lower plasma erythropoietin levels in some chronic hemodialysis patients; and sirolimus, an immunosuppressant agent that causes marrow suppression and anemia, can be used to help treat PTE (Charfeddine et al., 2008). To decrease the risk of thromboembolic events, intermittent venesection (or phlebotomy) should be performed in the PTE patients if their hemoglobin level cannot be adequately lowered with medications or their hematocrit values exceed 55% (Vlahakos et al., 2003).

## Post-transplant cytopenias (PTC)

Cytopenia is a disorder in which the production of one or more blood cell types ceases or is greatly reduced. This disorder is often caused by immunosuppressive therapy, chemotherapy, and viral infections after renal transplantation. Cytopenia include anemia (adeficiency of RBCs), leukopenia or neutropenia (a deficiency of WBCs or leukocytes), thrombocytopenia (a deficiency of platelets), and pancytopenia (a deficiency of all three blood cell types—RBC, WBC, and platelet) (Smith, 2010).

### Leukopenia or neutropenia

Leukopenia is a common occurrence following organ transplantation. It is defined as that a total WBC count is less than 3000–4000 cells/μL. The most common form of leukopenia is neutropenia which is an abnormally low count of neutrophils, and generally defined as a count of 1500 or fewer cells/μL (Smith et al., 2014). About 20–63% of kidney recipients will experience at least one episode of leukopenia/neutropenia. It typically occurs around day 100 after transplantation and can last from 1 to 4 weeks (Luan et al., 2011). The cause of leukopenia/neutropenia is usually multifactorial. The most predictable immunosuppressive agent to cause leukopenia/neutropenia is AZA, with 50% of renal transplant recipients developing leukopenia/neutropenia (Pollak et al., 1980). However, leukopenia/neutropenia due to AZA is most often reversible with decrease or discontinuation of the drug. T-cell depleting antibody therapies (thymoglobulin, atgam, alemtuzumab, and basiliximab) can result in some degree of leukopenia/neutropenia by eliminating targeted lymphocytes (Zaza et al., 2014). Leukopenia/neutropenia due to the use of MMF is seen in 13–35% of renal transplant recipients. The marrow effects of MMF are correlated with active metabolite, mycophenolic acid (MPA). The anti-CMV myelosuppressive agents (valganciclovir and ganciclovir) have been shown to cause leukopenia/neutropenia in 50% of transplant patients in a dose-dependent manner (Brum et al., 2008). The calcineurin inhibitors (CsA, FK-506, and sirolimus) are also reported to increase the risk of hematologic toxicity and leukopenia/neutropenia (De Rycke et al., 2011). Several commonly used antibiotics (such as trimethoprim-sulfamethoxazole, beta-lactam antibiotics, and piperacillin) can be frequently implicated as a possible etiology for leukopenia/neutropenia (Lee et al., 2009). Deficiencies of some essential nutrients, such as folic acid, vitamin B12, zinc, and copper, may also lead to leukopenia/neutropenia (Munshi and Montgomery, 2000). Viral infections have marked myelosuppression effects in renal transplant recipients, and have been reported to have leukopenia/neutropenia as a manifestation, including CMV, PVB19, herpesvirus-6 (HHV-6), influenza, and ehrlichiosis (Kotton and Fishman, 2005).

There are no ideal therapies for leukopenia/neutropenia after renal transplantation, but the most effective way to attenuate leukopenia/neutropenia is to discontinue the most likely offending drugs (such as MMF, valganciclovir, CsA, and FK-506) or cut down their doses. However, this strategy is frequently linked to increase risk of rejection. Recombinant granulocyte-colony stimulating factors (G-CSF), such as filgrastim (Neupogen), may be effective in treating leukopenia/neutropenia cuased by chemotherapy with valganciclovir or ganciclovir. G-CSF can be considered a second-line of therapy after adjustment of the other medications. In addition, stem cell transplants may be useful in treating some types of severe leukopenia/neutropenia, including those caused by the myelosuppressive agents (Hartmann et al., 2008).

### Thrombocytopenia

Thrombocytopenia is defined as that a total platelet count is less than 50,000/μL. It is quite prevalent in the first year after renal transplantation. Most renal recipients may show the lowest platelet count in the first 3 months. The main clinical manifestations of thrombocytopenia include the mild to serious bleeding, bruising, petechial, malaise, fatigue, and general weakness (Xie et al., 2013). Thrombocytopenia often occurs as a result of bone marrow suppression due to immunosuppressant agents, infection, acute rejection episodes, chemotherapy, antiplatelet antibody therapy, microangiopathy, or deficiencies of folate and Vitamin B12 (Najean et al., 1997). The cause of thrombocytopenia is usually overlapped with the causes of anemia and leukopenia in renal transplant recipients. After the use of sirolimus and/or calcineurin inhibitors, microangiopathy has been reported as a cause of thrombocytopenia in renal transplant recipients (Robson et al., 2003). Other medications that cause thrombocytopenia include rabbit antithymocyte globulin, valganciclovir, ganciclovir, linezolid, and heparin (Gabardi et al., 2004). Viral infections, particularly CMV or EBV infection, can cause thrombocytopenia and HPS (Torti et al., 2012).

The main concepts in treating thrombocytopenia are to discontinue suspected drugs that cause thrombocytopenia, to stimulate the bone marrow production of platelets, to maintain platelet at an adequate level, and to treat microangiopathy (Provan et al., 2010). In drug-induced thrombocytopenia, discontinuation or alternatives of suspected drugs will be helpful in treating thrombocytopenia. Corticosteroids may be used to increase platelet production. Lithium carbonate or folate may also be used to stimulate the bone marrow production of platelets. Blood (platelet) transfusions may be used to stop abnormal bleeding caused by thrombocytopenia. Rituximab, daclizumab, and other new antibody preparations may be effective for patients with transplant associated TMA. New generation thrombopoietin growth factors (such as romiplostim and eltrombopag) have been used as second line therapy of immune thrombocytopenia for hematopoietic stem cell transplant patients (Wörmann, 2013), and may be effective in treating post-transplant thrombocytopenia.

### Pancytopenia

Pancytopenia, the deficiency of all three blood cell types (RBCs, WBCs, and platelets), is characteristic of aplastic anemia, a potentially life-threatening disorder that requires a stem cell transplant. Pancytopenia has widespread effects on the entire body by leading to oxygen shortage as well as problems with immune function (Narang and Sachdeva, 2012). Pathologies involving the WBC and platelet population often exist in the context of pancytopenia, which can probably be a manifestation of systemic infection (Marinella, 2010). In renal transplant recipients, PVB19 infection is a common cause of pancytopenia and leads to various forms of glomerulopathy and allograft dysfunction (Yango et al., 2002). In addition, visceral leishmaniasis, a disease caused by protozoan parasites of the genus Leishmania and spread by the bite of certain types of sandflies, can also cause pancytopenia in some immunocompromised renal transplant recipients (Aardema et al., 2009). Other potential factors involved in the development of pancytopenia include immunosuppressive drugs (azathioprine, MPA, anti-thymocyte globulins, and alemtuzumab), chemotherapy drugs that cause bone marrow suppression, antibiotics (linezolid and chloramphenicol), and radiation therapy (Nagasawa et al., 2004). Symptoms of pancytopenia can include bleeding, bruising, fatigue, shortness of breath, and weakness. Treatments for pancytopenia include drugs that suppress the immune system, bone marrow stimulant drugs, blood transfusion, bone marrow transplant, and stem cell replacement therapy (Narang and Sachdeva, 2012).

## Other hematological complications of renal transplantation

Uncommon but serious hematologic complications in the renal transplant recipients include HPS, TMA, and therapy-related myelodysplasia (t-MDS) and t-AML (Marinella, 2010). In renal transplant recipients, the prognosis of these hematological complications is usually severe, with mortality occurring in more than 50% of patients (Mintzer et al., 2009).

### Hemophagocytic syndrome (HPS)

HPS, also referred as hemophagocytic lymphohistiocytosis (HLH) or macrophage activation syndrome (MAS), is a clinicopathologic entity characterized by uncontrolled proliferation of hematophagic monocytes/macrophages/histiocytes that are actively ingesting other blood cells. In most cases, HPS is associated with opportunistic infection following intensive immunosuppression. HPS cases are observed not only in association with viral infections [such as CMV, adenovirus, EBV, human herpes virus 8 (HHV-8), human herpes virus 6 (HHV-6), PVB19, and BK polyoma virus], but also with infections due to bacteria (such as tuberculosis, *Bartonella henselae*, and *Escherichia coli*) and protozoan (such as toxoplamosis, leishmaniasis, pneumocystis carinii pneumonia, and babebiosis) (Karras et al., 2004; Ponticelli and Alberighi, 2009). Pathogenesis of post-transplant HPS may involve (1) the activation of T helper-1 (Th-1) cells and the overproduction of cytokines, tumor necrosis factor-alpha (TNF-α) and interferon-gamma (IFN-γ) caused by severe infection, and (2) abnormalities of CD8 + T lymphocyte and natural killer-cell (NK-cell) cytotoxicity caused by immunosuppression. These cellular and biochemical alterations may lead to excessive Th-1 lymphocyte and macrophage activation and uncontrolled proliferation under lack of NK cell and T lymphocyte cytotoxicity, thus causing hemophagocytosis (Ponticelli and Alberighi, 2009).

HPS usually develops in the first few months (35–61 days) after renal transplantation, but in some recipients with parasitic infection or neoplasia it may occur years after transplantation. Generally, post-transplant HPS is associated with a higher rate (53%) of death in renal transplant recipients (Gurkan et al., 2006). Diagnostic criteria for HPS may include fever, cytopenia of two lines, hypertriglyceridemia, hypofibrinogenemia, hyperferritinemia (>500 μg/L), hemophagocytosis, elevated soluble interleukin-2 receptor (CD25), decreased NK-cell activity, and hepato-splenomegaly (Ponticelli and Alberighi, 2009).

The main goal of therapy for HPS is to recognize promptly and treat the etiological microorganism. Reduction or withdrawal of immunosuppressive agents is usually recommended in order to control infection. Intravenous methylprednisolone (Medrol) may attenuate hemophagocytosis by reducing the activation of macrophages and cytokines, but a prolonged administration may worsen the underlying infection (Karras et al., 2004). CMV infection could be treated with intravenous ganciclovir resorting to foscarnet or cidofovir. HHV-8 infection might be rescued by reduction of immunosuppression and the administration of foscarnet. BK virus infection may be treated by withdrawal of immunosuppressive therapy, infusion of intravenous IVIg and increasing prednisone. The use of IVIg may also be useful for treating bacteria and protozoan infections. In addition, graft nephrectomy may be a possible therapeutic option for renal transplant recipients with HPS resistant to standard supportive therapy (Gurkan et al., 2006).

### Thrombotic microangiopathy (TMA)

TMA is a group of disorders characterized by thrombocytopenia, MAHA (intravascular hemolysis and presence of peripheral blood schistocytes), purpura, microvascular occlusion (thrombi and coagulation) neurological symptoms, fever, and renal dysfunction. HUS and thrombotic thrombocytopenic purpura (TTP) are two major types of TMA (Barbour et al., 2012). TMA is also a distinct and severe complication of both renal transplantation and HSCT that are closely associated with calcineurin inhibitors and often cause graft failure (Laskin et al., 2011). The calcineurin inhibitors (CsA and FK-506) are directly toxic to microvascular endothelial cells and can induce microvascular constriction and platelet aggregation. Microvascular endothelial injury and platelet aggregation play an important role in the development of TMA in renal transplant recipients. Intravascular thrombi of aggregated platelets lead to thrombocytopenia and various degrees of organ ischemia and anemia. Furthermore, viral infections (CMV, HIV, and PVB19), severe renal ischemia and antibody-mediated acute humoral rejection may also be implicated in TMA (Laskin et al., 2011).

In renal transplant recipients, TMA may occur *de novo* (triggered by immunosuppressive drugs and acute antibody-mediated rejection) or recur in patients with previous history of HUS (Noris and Remuzzi, 2010). The majority of *de novo* TMA cases is related to calcineurin inhibitor therapy and occurs at a median of 25 days after transplantation. Clinical features of *de novo* TMA include anemia (hemoglobin <10 g/dL), thrombocytopenia, increased lactate dehydrogenase, decreased haptoglobin and schistocytes (Caires et al., 2012). Calcineurin inhibitor withdrawal or reduction should be the first step in the management of post-transplantation TMA. Patients with systemic TMA need to receive plasma therapy (fresh frozen plasma infusion or plasmapheresis), which is a procedure which replaces proteins necessary for the complement cascade that the patient does not have (Noris and Remuzzi, 2010). Some of these patients may also need dialysis therapy (Caires et al., 2012). The targeted complement C5 inhibitor therapy (eculizumab for atypical HUS and rituximab for TTP) is most likely to be effective in treating TMA (Barbour et al., 2012). Rituximab (with or without cyclophosphamide) may be efficacious as treatment for TTP (Laskin et al., 2011).

### Therapy-related myelodysplastic syndromes (t-MDS) and acute myeloid leukemia (t-AML)

t-MDS (also known as myelodysplasia) is a hematological medical condition with ineffective production of all blood cells. It is characterized by blood cytopenias, ineffective hematopoiesis, dyserythropoiesis, dysgranulopoiesis, dysmegakaropoiesis, and increased myeloblast. The median time to development of t-MDS is about 3–5 years (Bhatia, 2013). Patients with t-MDS often develop severe anemia, cytopenias and refractory AML. t-AML, also called acute myelogenous leukemia or acute non-lymphocytic leukemia (ANLL), is a disorder of the myeloid line of blood cells, characterized by the rapid growth of abnormal white blood cells that accumulate in the bone marrow and interfere with the production of normal blood cells. The average time interval between onset of t-AML and receiving organ transplantation is about 5 years. The symptoms of t-AML include fatigue, shortness of breath, easy bruising and bleeding, petechiae, bone and joint pain, and persistent or frequent infections (such as gram-negative septicemia). t-AML progresses rapidly and the patients may die within weeks or months if left untreated (Thalhammer-Scherrer et al., 1999). Both t-MDS and t-AML are two treatment-related complications that occur in organ transplant recipients treated with immunosuppressive agents. The two conditions are often associated with heavy post-transplant immunosuppression by azathioprine (a thiopurine prodrug) (Rashidi and Fisher, 2014) or by ATG (Naithani et al., 2005). The ability of azathioprine to promote clonal expansion of DNA mismatch repair (MMR)-deficient myeloid cells is consistent with a role in the development of t-MDS/t-AML in patients who have received organ transplants (Bhatia, 2013).

Genetic variation in susceptibility to genotoxic exposures [such as the use of chemotherapy agents (alkylating drugs), epipodophyllotoxins and anthracyclines] increases significantly the risk for development of t-MDS/t-AML. Chromosome 7 and/or 5 losses or deletions that are caused by alkylating chemotherapy agents are related to the development of t-MDS/t-AML. Variation on chromosome 7 and 5 may cause translocations of chromosome bands 11q23 and 21q22 to encode abnormal fusion proteins (transcription factors) and inhibit myeloid differentiation(Bhatia, 2013). Furthermore, epigenetic changes in DNA structure have been considered as a mechanism of t-MDS/t-AML. The loss of DNA methylation control due to a “differentiation arrest” in myeloid cells may lead to uncontrolled growth of an immature clone of cells, thus developing t-MDS/t-AML (Graubert and Walter, 2011; Larsson et al., 2013; Mazzarella et al., 2014). Other genetic, pharmacological and radiotherapeutic factors, such as predisposing factors (polymorphisms in detoxification and DNA repair enzymes), granulocyte-colony-stimulating factor, topoisomerase II inhibitors, and radiotherapy, may cause chromosome abnormalities (specifically del 5q, −7, +8, and del 20q) of bone marrow cells. These factors also play a significant role in induction of t-MDS/t-AML (Leone et al., 2007; Sebaa et al., 2012).

Discontinuing the use of azathioprine (Offman et al., 2004) or replacing azathioprine with a nonthiopurine alternative (such as mycophenolate, sirolimus, or everolimus) (Bhatia, 2013), has been proven to reduce the incidence of post-transplantation t-MDS/t-AML. Three DNA methyltransferase inhibitors (5-azacytidine, decitabine, and lenalidomide) approved by the US FDA for the treatment of t-MDS, can restore normal blood counts and retard the progression of MDS to acute leukemia by creating a more orderly DNA methylation profile in the hematopoietic stem cell nucleus (Gore and Hermes-DeSantis, 2008). Supportive care with blood product support (RBC transfusion), iron chelators (deferoxamine and deferasirox), and hematopoeitic growth factors (erythropoietin), is the mainstay of therapy for t-MDS/t-AM (Greenberg et al., 2011). Chemotherapy with the hypomethylating agents (5-azacytidine and decitabine) may help slow the progression of MDS to AML (Garcia et al., 2010). Treatment for AML is usually divided into two phases: induction and consolidation therapy. Induction therapy with cytarabine (Ara-C) and anthracylines may achieve a complete remission by reducing the number of leukemic cells to an undetectable level. Consolidation therapy is the intensive chemotherapy to eliminate any residual disease (Leahey et al., 1999). After the completion of consolidation therapy, a combination immunotherapy with histamine dihydrochloride (INN) and interleukin 2 can markedly reduce the risk of relapse in AML and maintain remission of symptoms (Romero et al., 2009). For patients with relapsed t-AML or t-MDS, HSCT can be considered as a potentially curative therapeutic option (Kekre and Koreth, 2015).

## Conclusions

Blood disorders after renal transplantation are frequently observed. PTA, PTLD, PTE, and PTC are common hematological complications, and HPS, TMA and t-MDS/t-AML are uncommon but serious hematological complications in renal transplant recipients. Multiple factors, including immunosuppressive drugs, allograft dysfunction, viral or bacterial infections, antibiotics, chemotherapy, decreased or increased EPO production, transplant rejection episodes and nutritional deficiencies, are associated with the development of post-transplant blood disorders. Of these etiological factors, chronical exposure to immunosuppressive agents and subsequent viral infections can possibly be the two major contributors to the post-transplant blood disorders. Bone marrow suppression, which is typically due to the direct effects of both immunosuppressive agents and viral infections, is a fundamental cause of most blood disorders in renal transplant recipients (Table 1 in Supplementary Material). A series of molecular biological events, including genetic or epigenetic alterations of cellular genes (DNA mutations, DNA hypermethylation, or polymorphisms in DNA), DNA mismatch repair defect, inhibition of hematopoietic stem cell proliferation, and chromosome abnormalities, have been proven to involve the development of the drug or virus-induced bone marrow suppression. As the direct consequences of myelosuppression, hematological abnormalities, and immunodeficiency will cause severe clinical outcomes (such as systemic infection symptoms, cardiovascular diseases, renal allograft dysfunction or loss, CKD or CAN, and death) in patients with post-transplant blood disorders. Pharmacological treatment strategies for post-transplant blood disorders should aim at tapering immunosuppressive therapy or replacing myelotoxic immunosuppressive drugs with lower toxic alternatives (e.g., ASTRAGRAF XL), recognizing and treating promptly the etiological microorganism, attenuating bone marrow suppression, restoring normal blood counts, retarding the progression of a blood disorder, enhancing kidney graft survival, and treating underlying complications. Currently, there is an urgent need to develop a new generation of immunosuppressant agents that don't cause myelotoxicity in renal transplant recipients. New immunosuppressant drugs without myelotoxicity will have the potential to decrease the incidence of blood disorders following renal transplantation and to improve long-term kidney graft survival. HSCT can be considered as a potentially curative therapeutic option for some types of blood disorders.

### Conflict of interest statement

The authors declare that the research was conducted in the absence of any commercial or financial relationships that could be construed as a potential conflict of interest.
